# Diode Laser Detection of Greenhouse Gases in the Near-Infrared Region by Wavelength Modulation Spectroscopy: Pressure Dependence of the Detection Sensitivity

**DOI:** 10.3390/s100504686

**Published:** 2010-05-06

**Authors:** Takashi Asakawa, Nozomu Kanno, Kenichi Tonokura

**Affiliations:** 1 Department of Chemical System Engineering, The University of Tokyo, 7-3-1 Hongo, Bunkyo-ku, Tokyo 113-8656, Japan; E-Mail: asakawa@esc.u-tokyo.ac.jp; 2 Department of Micro-Nano Systems Engineering, Nagoya University, Furo-cho, Chikusa-ku, Nagoya 464-8603, Japan; E-Mail: kanno@yoshilab.nuae.nagoya-u.ac.jp; 3 Environmental Science Center, The University of Tokyo, 7-3-1 Hongo, Bunkyo-ku, Tokyo 113-0033, Japan

**Keywords:** gas sensing, wavelength modulation spectroscopy, near-infrared absorption spectroscopy

## Abstract

We have investigated the pressure dependence of the detection sensitivity of CO_2_, N_2_O and CH_4_ using wavelength modulation spectroscopy (WMS) with distributed feed-back diode lasers in the near infrared region. The spectral line shapes and the background noise of the second harmonics (2*f*) detection of the WMS were analyzed theoretically. We determined the optimum pressure conditions in the detection of CO_2_, N_2_O and CH_4_, by taking into consideration the background noise in the WMS. At the optimum total pressure for the detection of CO_2_, N_2_O and CH_4_, the limits of detection in the present system were determined.

## 1. Introduction

Atmospheric pollution is a problem as global warming is caused by the man-made addition of extra amounts of greenhouse gases such as CO_2_, N_2_O and CH_4_. Current average mixing ratios of the trace components CO_2_, N_2_O and CH_4_ in the atmosphere are ∼380, ∼1.8 and ∼0.32 parts per million by volume (ppmv), respectively. CO_2_ is released into the atmosphere when fossil fuels, coal, solid waste and wood products are burned, during cement production, and also when land surface cover is changed by humans. Increased CH_4_ levels come from fossil fuels, rice cultivation, animal husbandry, biomass burning and landfills. The main anthropogenic sources of N_2_O are agriculture, and industrial sources including adipic and nitric acid production. Combustion of solid waste and fossil fuels also contributes to atmospheric N_2_O. The need to detect these trace gases has become increasingly important in recent years, both for controlling industrial processes and for monitoring air quality. Since many trace gases have a significant impact on the environment, the development of techniques for its fast, accurate and sensitive detection is required [1–5].

Laser based spectroscopy provides interesting advantages related to its high selectivity and sensitivity in the detection of trace gases. Semiconductor diode lasers emitted the near infrared (NIR) light have played a central role, being tunable spectroscopic light sources which exhibit a relatively low amplitude noise [6]. In addition, their small size and the relatively low cost have made them particularly suitable for the realization of transportable spectrometers employed for *in-situ* measurements [7,8]. In the NIR region, relatively weak overtone or combination vibrational transitions occur for trace gases relevant to the atmospheric environment. Although line strengths of these transitions are a few orders of magnitude lower than transitions belonging to fundamental vibrational bands, cavity ring down spectroscopy (CRDS) [9] and frequency modulation spectroscopy (FMS) with a single-mode diode laser in the near infrared region have allowed to achieve the detection of low concentrations (< sub ppmv) of trace gases [10]. The use of multi-pass absorption cells [11] further improves the sensitivity, since the effective optical path is increased up to several tens or hundreds of meters. Phase-sensitive techniques, which are the core of the FM techniques, significantly reduces the 1/*f* electronic noise, achieving high detection sensitivity. In most cases the detection sensitivity is only limited by noise introduced by undesired optical fringes and drifting of laser power and detector sensitivity. Wavelength modulation spectroscopy (WMS) [12–16] is a kind of FMS with a modulation frequency lower than the spectral line width of interest. In the case of WMS, the lower modulation frequency allows one to use low-frequency circuits and photo detectors, reducing the complexity and the cost of measurement system. Recently, a high sensitive open path CH_4_ analyzer based on WMS detection and Herriott cell design with a 30 m total optical path length has been developed which is commercially available [17].

In the present study, we demonstrate the detection of greenhouse gases such as CO_2_, CH_4_ and N_2_O using WMS. The aim of this work is characterized the optimum condition of the detection of greenhouse gases by NIR-WMS. The optimum pressure for the detection of CO_2_, N_2_O and CH_4_ by WMS is investigated both experimentally and theoretically by taking into consideration background noise in the WMS.

## 2. Experimental

**Figure 1.**
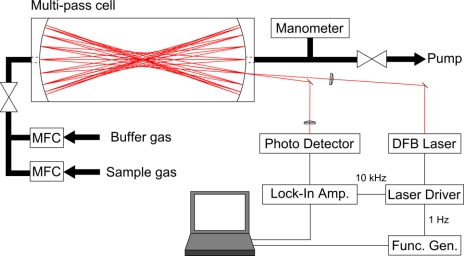
Schematic diagram of the experimental setup (MFC: mass flow controller. Func. Gen.: function generator).

We used WMS in the near-infrared region for trace gas detection. Figure 1 shows a scheme of our experimental apparatus. All optical elements were properly designed to reduce their dimensions and mechanical instability as much as possible. Three distributed feed-back (DFB) diode lasers (NTT Electronics) with a tuning range of ± 1 nm were used as light sources. The first DFB laser with a center wavelength of 1,572 nm was used to detect CO_2_ at the 3ν_1_ + ν_3_ combination band. The second DFB laser with a center wavelength of 1,515 nm was used to detect the N_2_O line at the 3ν_3_ overtone band. The last DFB laser with a center wavelength of 1,651 nm was used to detect CH_4_ at the 2ν_3_ overtone band. The laser power varied as a function of injection current with a maximum of 20 mW. The wavelengths of these DFB lasers were chosen for strong absorbing transitions and no interference of water.

The laser wavelengths were tuned by varying the laser temperatures, while fine tuning was accomplished by changing the laser diode injection currents. To minimize fringe noise from optics, anti-reflection (AR) coated lenses and AR coated and wedged windows were used. The output beam was focused by an AR coated lens (f = 50 cm) to the centre of a Herriott-type multi-pass cell [11] with a distance of 40.4 cm between two mirrors. In the multi-pass cell, the laser beam undergoes multiple reflections between two mirrors; the total number of 74 passes corresponds to the optical path length of 29.91 m. The inner volume of the cell is 900 cm^3^. The beam passed through the multi-pass cell was finally focused by a short focal AR coated lens (f = 5 cm) onto an InGaAs photodiode detector (Hamamatsu G5852-11).

The laser wavelength was sinusoidally modulated at 10 kHz, and scanned at 1 Hz around the absorption line. The laser wavelength scan and modulation were performed through a custom-made laser driver. The background bias signal of the laser driver used in this study was same level as that of a commercial driver (e.g., ILX LDC3724C). Under typical conditions, the modulated absorption signal was phase-sensitive detection at twice the modulation frequency (2*f*) using a lock-in amplifier (Stanford Research System SR810 DSP) with the time constant set to 3 ms. The data was acquired via a 16 bit AD PCMCIA card (CONTEC ADA16-8/2(CB)L) to a laptop computer and analyzed using software written in LabVIEW.

N_2_ (99.99%) and premixed gases of synthesized air (79% N_2_, 21% O_2_), 1% CO_2_ in N_2_, 1% N_2_O in N_2_, and 0.1% CH_4_ in air were purchased from Taiyo Nippon Sanso Co. The flow rate of the feed was controlled with mass flow controllers (Kofloc MODEL3660). The total pressure was monitored with a capacitance manometer (Setra Systems MODEL720). All measurements were performed at room temperature (293 ± 3 K).

## 3. WMS Theory

The general theory of the FMS and WMS has been well summarized in the works of previous researchers [13–15]. In the case of sinusoidal modulation with modulation index *β* and frequency *ω*_m_, the optical field *E* with carrier frequency *ω*_0_ is given by:

(1) $$E\left(t\right)=E_{0}\;\text{exp}\left[i\left(\omega_{0}t+\beta \;\text{sin}\;\omega_{m}t\right)\right]=E_{0}\;\text{exp}\left(i\omega_{0}t\right)\sum_{n=-\infty }^{+\infty }J_{n}\left(\beta \right)\text{exp}\left(\mathrm{in}\;\omega_{m}t\right)$$

where the expansion in a series of *n*th-order Bessel functions *J_n_* characterizes the frequency components of the modulated light spectrum. FMS is classified by its modulation frequency *ω*_m_. WMS uses *ω*_m_ that is much less than the spectral line width *Γ* of the absorption line of interest. Conversely, narrowly-defined FMS uses *ω*_m_ ≫ *Γ*. In the case of WMS, the probe beam modulation is generally treated as instantaneous frequency change:

(2) $$\omega_{i}\left(t\right)\equiv \frac{d}{dt}\left(\omega_{0}t+\beta \;\text{sin}\;\omega_{m}t\right)=\omega_{0}+\Delta F\;\text{cos}\;\omega_{m}t$$

where the maximum frequency deviation from the carrier frequency *ω*_0_ is Δ*F* = *βω*_m_. In this model, dispersion effects cannot be considered, and the light intensity transmitted through the sample is expressed by *I*_T_ = *I*_0_ exp(−*α*), where *α* is the absorption coefficient.

On the assumption that *α*(*ω*) ≪ 1, *I*_T_(*ω*) ≈ *I*_0_(*ω*)[1 − *α*(*ω*)], the *α*(*ω*) can be expanded into the Taylor series around the carrier frequency *ω*_0_:

(3) $$\begin{array}{c} I_{T}\left(\omega \right)=I_{0}\lfloor 1-a\left(\omega_{0}\right)-{\frac{d\alpha }{d\omega }|}_{\omega_{0}}\left(\omega -\omega_{0}\right)-\frac{1}{2!}{\frac{d^{2}\alpha }{d\omega^{2}}|}_{\omega_{0}}{\left(\omega -\omega_{0}\right)}^{2}\\ -\frac{1}{3!}{\frac{d^{3}\alpha }{d\omega^{3}}|}_{\omega_{0}}{\left(\omega -\omega_{0}\right)}^{3}-\ldots ]\\ =I_{0}\lfloor 1-a\left(\omega_{0}\right)-{\frac{d\alpha }{d\omega }|}_{\omega_{0}}\Delta F\;\text{cos}\;\omega_{m}t-\frac{1}{2!}{\frac{d^{2}\alpha }{d\omega^{2}}|}_{\omega_{0}}{\left(\Delta F\right)}^{2}\frac{1}{2}\left(1+\;\text{cos}\;2\omega_{m}t\right)\\ -\frac{1}{3!}{\frac{d^{3}\alpha }{d\omega^{3}}|}_{\omega_{0}}{\left(\Delta F\right)}^{3}\frac{1}{4}\left(3\;\text{cos}\;\omega_{m}t+\;\text{cos}\;3\omega_{m}t\right)\ldots ]. \end{array}$$

Here cos^2^ *ω*_m_*t*, cos^3^ *ω*_m_*t*, *etc*., have been expanded with trigonometric identities for convenience in picking off the power at any frequency of interest. Because the instantaneous frequency varies sinusoidally, the Taylor series of *I*_T_(*ω*) is more or less automatically the Fourier series, too. This series will not converge well for some Δ*F* [16]. However, if the frequency deviation Δ*F* is sufficiently small compared with *Γ*, higher-order terms can be neglected, and the WMS signal that is coherently detected at the frequency *nω*_m_ is proportional to the *n*-th derivative of the sample absorption.

In the present study, the assumption that Δ*F* ≪ *Γ* was not valid under some experimental conditions. Thus we used the frequency domain approach to calculate the WMS spectral line shape. In the real system, the DFB laser intensity is not uniform over the FM range, and simultaneous amplitude modulation (AM) of the electric field occurs such that:

(4) $$E(t)=E_{0}\left[1+M\;\text{sin}\;\left(\omega_{m}t+\psi \right)\right]\text{exp}\left[i\omega_{0}t+i\beta \;\text{sin}\;\left(\omega_{m}t\right)\right]$$

where *M* and *ψ* denote the AM index and the phase difference between AM and FM. By expanding the AM term in term of exponentials, *i.e.*, *M*sin(*ω*_m_*t* + *ψ*) = *M*/2[exp{*i*(*ω*_m_*t* + *ψ*)} − exp{*i*(*ω*_m_*t* + *ψ*)}], and replacing the exponential frequency terms as a summation over Bessel functions, one can rewrite Equation (4) as:

(5) $$E\left(t\right)=E_{0}\;\text{exp}\left(i\omega_{0}t\right)\sum_{n=-\infty }^{\infty }r_{n}\;\text{exp}\left({\mathrm{in}\;\omega }_{m}t\right)$$

where:

(6) $$r_{n}=\sum_{k=-1}^{1}a_{k}J_{n-k}\left(\beta \right),\;\;\;\;\;\;\;\;\;\;a_{0}=1,a_{\pm 1}=\frac{\pm M}{2i}\text{exp}\left(\pm i\psi \right)$$

Equation (1) is the special case of Equation (5) where *M* = 0.

When the modulated electric field *E*(*t*) is passed through a medium exhibiting the field amplitude attenuation *δ*(*ω*) and the optical phase shift *φ*(*ω*), the transmitted optical field is then:

(7) $$E_{T}\left(t\right)=E_{0}\;\text{exp}\left(i\omega_{0}t\right)\sum_{n=-\infty }^{\infty }\text{exp}\left(-\delta_{n}-i\varphi_{n}\right)r_{n}\;\text{exp}\left(\mathrm{in}\;\omega_{m}t\right)$$

where *δ_n_* = *δ*(*ω*_0_ + *nω*_m_) and *φ*_n_ = *φ*(*ω*_0_ + *nω*_m_). Because the intensity *I* equals *cɛ*_0_EE*/2 in SI units, the absorption coefficient *α* is twice the electric field amplitude attenuation coefficient *δ* for weak absorption. The transmitted intensity can be expressed as a sum of Fourier components at frequencies corresponding to multiples of *ω*_m_ [13]. In the case of 2*ω*_m_ detection, the resulting signal is:

(8) $$I={2I}_{0}\left[\text{Re}\left(Z\right)\text{cos}\;\theta -\text{Im}\left(Z\right)\text{sin}\;\theta \right],\;\;\;\;Z=\sum_{n=-\infty }^{\infty }r_{n+1}r_{n-1}^{*}\left[\text{exp}\left(-\left(\delta_{n+1}+\delta_{n-1}\right)\right)+i\left(\varphi_{n+1}-\varphi_{n-1}\right)\right]$$

where *θ* refers to the phase difference between the transmitted intensity and the reference signal in the lock-in amplifier. The in-phase (0 or π) component corresponds to absorption, and the quadrature (±π/2) components correspond to dispersion of the incident light. In the absence of absorption and dispersion, *Z* has a nonzero value due to residual amplitude modulation (RAM) [14]. In the case of 2*ω*_m_ detection, the background bias signal is:

(9) $$I_{\text{RAM}}=\frac{1}{2}M^{2}I_{0}\;\text{cos}\left(\theta +2\psi +\pi \right)$$

Since *I*_RAM_ linearly depends on *I*_0_, low frequency noise in *I*_0_, such as fringe noise from optics and drifting of laser power and detector sensitivity, within the noise equivalent bandwidth of the lock-in amplifier is converted into FM/WM signal background noise.

**Figure 2.**
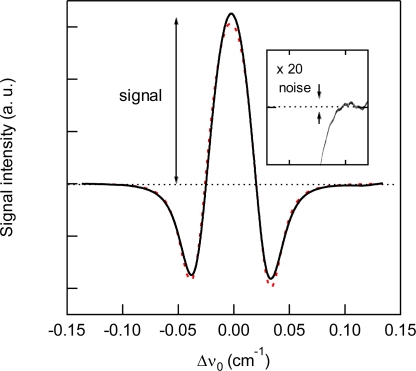
Experimental (solid line) and simulated (dashed line) 2*f* wavelength modulation spectra of 1% CO_2_ at around 6,359.97 cm^−1^ and a total pressure of 15 kPa diluted by N_2_.

WM spectra are calculated from Equation (8) assuming that the absorption peak shape is described by the Voigt function. The Voigt function was calculated following the algorithm described by Humlicek [18] and Schreier [19]. The Doppler half width at half maximum (HWHM) was calculated from the temperature and molecular weight. To calculate homogeneous HWHM values, the pressure broadening factor was taken from the HITRAN database [20]. The FM index *β* was estimated from *β* = Δ*F*/*ω*_m_. To calculate the AM index *M*, the intensity dependence on the laser frequency was measured, separate from the WMS experiments. Table 1 shows the parameters used in the spectral simulation. For the modulation frequency below 750 MHz, the AM-FM phase difference *ψ* has been found to have a value of ca. *n*π + π/2 [14]. For the present study, negative dependence of the laser intensity on the laser frequency suggests that *ψ* ≈ −π/2. In the WMS condition, *i.e.*, *ω*_m_ ≪ *Γ*, the dispersion term is negligibly smaller than the absorption term, thus the detection phase *θ* ≈ 0 for the maximum signal intensity. In all calculations, the values of *ψ* and *θ* were fixed to be –π/2 and 0, respectively. The signal intensity and noise are defined as peak-to-baseline amplitude and peak-to-peak variation of 2f signal with standard deviation of baseline, as shown in Figure 2.

## 4. Results and Discussion

**Figure 3.**
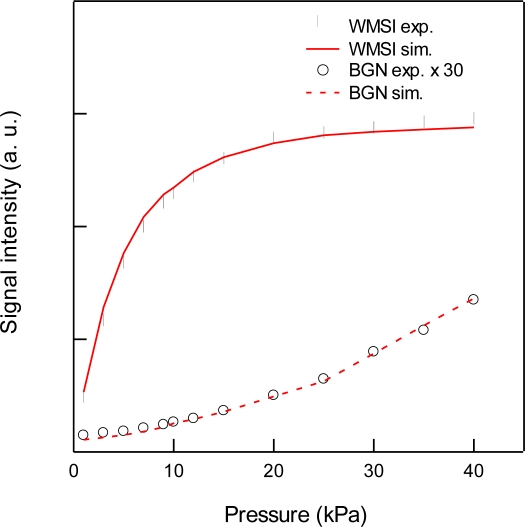
Total pressure dependence of 2*f* wavelength modulation signal intensities (WMSI; •, 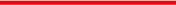) and background noise (BGN; ○, 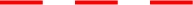) for 1% CO_2_ at 6,359.97 cm^−1^ diluted by N_2_. Circles and lines indicate experimental and simulated results, respectively.

**Figure 4.**
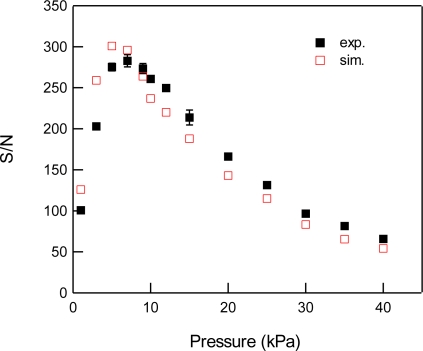
Total pressure dependence of S/N for 1% CO_2_ at 6,359.97 cm^−1^ diluted by N_2_. Closed and open squares indicate experimental and simulated results, respectively.

For the detection of CO_2_, we used the 3ν_1_ + ν_3_ combination band of the R(16) line at the absorption line center of 6,359.97 cm^−1^, which is free from interference by the other atmospheric species and has a line strength of 1.73 × 10^−23^ cm^2^ molecule^−1^ cm^−1^, according to the HITRAN database [20]. Figure 2 shows experimental and simulated spectra of 1% CO_2_ at a total pressure of 15 kPa, diluted by N_2_. The experimental spectra were acquired with a lock-in time constant of 3 ms and filter slope of 24 dB/oct, which leads a noise equivalent bandwidth of the low pass filter to be 26 Hz. The simulated spectrum reconstructed the experimental spectrum well. Figure 3 shows the total pressure dependence of signal intensity and background noise. In all experiments, the modulation depth Δ*F* were adjusted to obtain the maximum signal intensity. The concentration of CO_2_ was maintained at 1% in N_2_. In the simulation, we assumed that background noise was linearly dependent on *M*^2^, according Equation (9), and the simulated noise intensities were normalized to minimize the residual to experimental values. As can be seen, the simulated results closely agree with the experimental measurements. The signal intensity profile of Figure 3 shows two opposing effects of pressure: decreasing spectral line centre intensities due to pressure broadening and an increasing absorber number density with increasing pressure. At low pressures (Doppler regime), the signal intensity increases linearly with pressure, while at high pressures (Lorentzian regime) the signal intensity become almost constant due to the canceling of two opposing terms with pressure. Figure 4 shows experimental and simulated profiles of the signal to noise ratio (S/N) as a function of total pressure. The optimum total pressure in the detection of CO_2_ was found to be around 7 kPa. In the WMS, optimization of the signal intensity occurs at Δ*F*/HWHM = 2.2 in the Voigt function to calculate the properties of the 2*f* line shape [21].

Because the Voigt HWHM is increased by pressure broadening with increasing pressure, the frequency deviation Δ*F* must be increased in order to maximize the WMS signal with increasing pressure. Since the AM index *M* depends linearly on Δ*F*, *i.e.*, *M*/*ω*_m_*β* (= *M*/Δ*F*) is constant, and the residual amplitude modulation *I*_RAM_ is linearly dependent on *M*^2^ according to Equation (9), the low frequency noise in *I*_0_ such as the fringe noise from optics and drifting of laser power and detector sensitivity was enlarged by factor of *M*^2^ at higher pressure, as shown in Figure 3.

**Figure 5.**
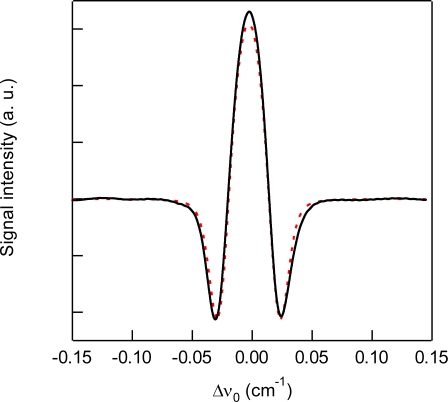
Experimental (solid line) and simulated (dashed line) 2*f* wavelength modulation spectra of 1% N_2_O at around 6,591.44 cm^−1^ and a total pressure of 6 kPa diluted by N_2_.

**Figure 6.**
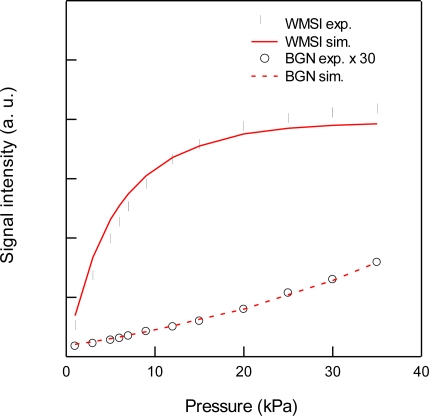
Total pressure dependence of 2*f* wavelength modulation signal intensities (WMSI; •, 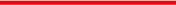) and back ground noise (BGN; ○, 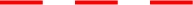) for 1% N_2_O at 6,591.44 cm^−1^ diluted by N_2_. Circles and lines indicate experimental and simulated results, respectively.

**Figure 7.**
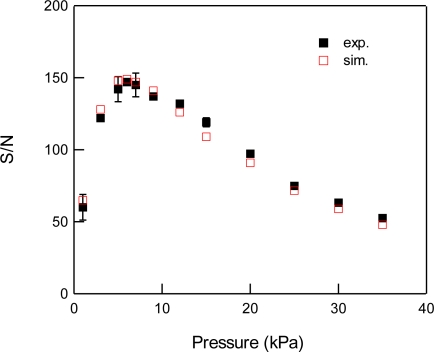
Total pressure dependence of S/N for 1% N_2_O at 6,591.44 cm^−1^ diluted by N_2_. Closed and open squares indicate experimental and simulated results, respectively.

In the case of N_2_O detection, the 3ν_3_ overtone band of the R(15) absorption line at 6,591.44 cm^−1^ with a line strength of 2.33 × 10^−23^ cm^2^ molecule^−1^ cm^−1^ was monitored. Figure 5 shows the experimental and simulated spectra of 1% N_2_O at a total pressure of 6 kPa. As for the previous experiment of CO_2_, the acquired 2*f* WM spectrum of N_2_O shown in Figure 5 is in excellent agreement with the simulated spectrum. Figure 6 shows the total pressure dependence of signal intensity and background noise for the 1 % N_2_O diluted in N_2_. The signal intensity is saturated above 20 kPa. Figure 7 shows experimental and simulated profiles of S/N as a function of total pressure. The simulated results agreed with the experimental results. The optimum total pressure for the detection of N_2_O is estimated to be around 6 kPa.

**Figure 8.**
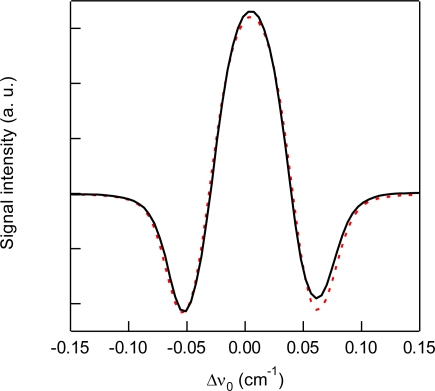
Experimental (solid line) and simulated (dashed line) 2*f* wavelength modulation spectra of 0.01% CH_4_ at around 6,046.95 cm^−1^ and a total pressure of 15 kPa diluted by N_2_.

**Figure 9.**
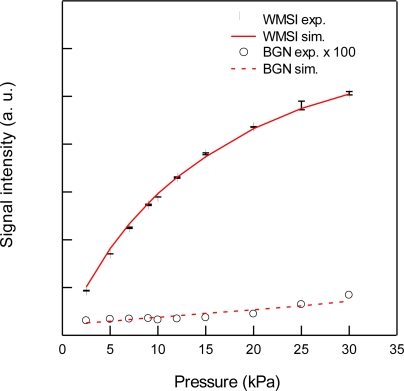
Total pressure dependence of 2*f* wavelength modulation signal intensities (WMSI; •, 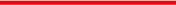) and background noise (BGN; ○, 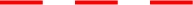) for 0.01% CH_4_ at 6,046.95 cm^−1^ diluted by N_2_. Circles and lines indicate experimental and simulated results, respectively.

**Figure 10.**
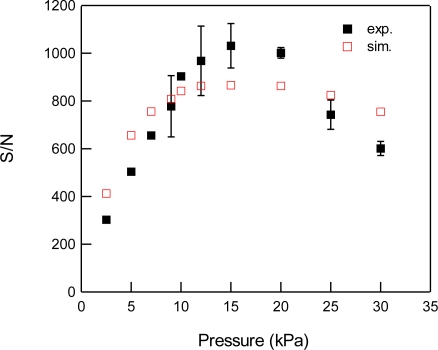
Total pressure dependence of S/N for 0.01% CH_4_ at 6,046.97 cm^−1^ diluted by N_2_. Closed and open squares indicate experimental and simulated results, respectively.

For the detection of CH_4_, we used the R(3) lines of the 2ν_3_ overtone band around 6,046.95 cm^−1^ with a line strength of the order 10^−21^ cm^2^ molecule^−1^ cm^−1^ [20]. As listed in Table 2, there are three absorption peaks at around 6,046.95 cm^−1^. Figure 8 shows the experimental and simulated spectra of 0.01% CH_4_ at around 6,046.95 cm^−1^ and total pressure of 10 kPa. The three absorption lines were considered in the simulation. In the experimental spectra, only one absorption peak was observed at 6,046.95 cm^−1^ due to the overlapping of the three absorptions. Figure 9 shows the total pressure dependence of signal intensity and noise for the 0.01 % CH_4_ diluted in N_2_. Figure 10 shows the experimental and simulated profiles of S/N as a function of total pressure. The simulated spectrum using the three absorption lines reproduces the experimental results well. The optimum total pressure of CH_4_ detection at the 6,046.95 cm^−1^ absorption peak was found to be around 15 kPa. The larger optimum pressure for CH_4_ detection compared with those for CO_2_ and N_2_O would be caused by the broader nature of the absorption peak consisting of three closely separated absorption lines.

To estimate the limit of detection (LOD) of the present system, we changed the mixing ratio of sample gases, while keeping the optimum value of the total pressure fixed. The measurements were performed with a noise equivalent bandwidth of 26 Hz. From a fit of the signal intensity as a function of CO_2_ mixing ratio at 7 kPa, we found, for a signal to noise ratio of 2, the LOD of [CO_2_]^7kPa^ = 24 ± 2 ppmv. In the case of N_2_O, the LOD at 6 kPa was estimated to be 7.6 ± 0.4 ppmv. For CH_4_, we estimated the detection limit and NEAS using the R(3) peaks at 15 kPa to be 60 ± 4 ppbv. The LODs for the three gases had an almost linear dependence on the absorption cross sections. The line strengths of CO_2_, N_2_O and CH_4_ of the used transitions vary slightly with temperature (0.23, 0.25 and 0.39%, respectively, by temperature change of 1 K around room temperature), however, the LODs are unaffected by temperature change. Parkes *et al.* demonstrated the detection of N_2_O by diode laser cavity ring-down spectroscopy using the same 3ν_3_ overtone band [22]. The limiting sensitivity was 23 ppmv at 1 atm with mirror reflectivities of R = 0.9998, which is comparable with our 2*f* WMS value of 7.6 ppmv. The noise-equivalent absorption sensitivity (NEAS) per scan for a signal to noise ratio of 1 was estimated using following Equation (10):

(10) $$\mathrm{NEAS}=\frac{{\mathrm{Abs}}_{\text{min}}}{l\;\sqrt{\mathrm{NEBW}/N}}$$

where *Abs*_min_, *l*, *NEBW* and *N* denote the absorbance at S/N = 1, the effective optical length of 29.91 m, the noise equivalent bandwidth of 26 Hz and the average number of 20 times, respectively. In the calculation of *Abs*_min_, the absorption line shapes were assumed to be described as the Voigt functions. The estimated values of NEAS for CO_2_, N_2_O and CH_4_ were found to be (1.3 ± 0.1) × 10^−8^, (4.8 ± 0.3) × 10^−9^, and (5.6 ± 0.4) × 10^−9^ cm^−1^ Hz^−1/2^, respectively. The higher value of NEAS for CO_2_ would be caused by the larger value of *M*/*ω*_m_*β*, as shown in Table 1, which results the larger effect of the residual amplitude modulation.

## 5. Conclusions

We have described the highly sensitive detection of CO_2_, N_2_O and CH_4_ by 2*f* WMS, using tunable near-infrared diode lasers in the near-infrared region. The observed 2*f* WM spectra were analyzed as a function of the total pressure. At the optimum total pressure for the detection of each gas, as determined by experimental and theoretical approaches, the limits of detection in the present system were determined. Detection of atmospheric N_2_O (320 ppbv) at the 3ν_3_ overtone band is not sufficient with our current WMS spectrometer. However, the necessary sensitivity could be delivered by reducing the optical fringe noise and the drifting of laser power using external modulators instead of direct laser modulation.

## Figures and Tables

**Table 1.** Parameters used in the spectral simulation.

**Laser**	**Target**	***ω*_0_*^a^***	***Γ*_Doppler_*^a^***	***Γ*_air_*^b^***	***Γ*_self_*^b^***	***M*/*ω*_m_*β^c^***
1	CO_2_	6,359.967	0.00593	0.0734	0.1009	0.423
2	N_2_O	6,591.437	0.00614	0.0774	0.1009	0.305
3	CH_4_	6,046.953	0.00933	0.0627	0.0820	0.288

a In units of cm^−1^.

b In units of cm^−1^/atm.

c In units of 1/cm^−1^.

**Table 2.** Absorption lines of R(3) of the CH_4_ 2ν_3_ band at around 6,046.97 cm^−1^.

**Line**	**Position (cm^−1^)**	**Integrated absorption cross-section (cm^2^ molecule^−1^ cm^−1^)**
1	6,046.942	8.17 × 10^−22^
2	6,046.953	1.00 × 10^−21^
3	6,046.965	1.32 × 10^−21^

## References

[1] Richter D., Fried A., Wert B.P., Walega J.G., Tittel F.K. (2002). Development of a tunable mid-IR difference frequency laser source for highly sensitive airborne trace gas detection. Appl. Phys. B.

[2] Gianfrani L., Sasso A., Tino G.M. (1997). Monitoring of O_2_ and NO_2_ using tunable diode lasers in the near-infrared region. Sens. Actuat. B.

[3] Uehara K., Yamamoto K., Kikugawa T., Yoshida N. (2001). Isotope analysis of environmental substances by a new laser-spectroscopic method utilizing different path lengths. Sens. Actuat. B.

[4] Hennig O., Strzoda R., Magori E., Chemisky E., Tump C., Fleischer M., Meixner H., Eisele I. (2003). Hand-held unit for simultaneous detection of methane and ethane based on NIR-absorption spectroscopy. Sens. Actuat. B.

[5] Katchalski T., Soria S., Teitelbaum E., Friesem A.A., Marowsky G. (2005). Two photon fluorescence sensors based on resonant grating waveguide structures. Sens. Actuat. B.

[6] Werle P. (1998). A Review of recent advances in semiconductor laser based gas monitors. Spectrochim. Acta A.

[7] Linnerud I., Kaspersen P., Jager T. (1998). Gas monitoring in the process industry using diode laser spectroscopy. Appl. Phys. B.

[8] Werle P., Mucke R., D’Amato F., Lancai T. (1998). Near-infrared trace-gas sensors based on room-temperature diode lasers. Appl. Phys. B.

[9] Crosson E.R. (2008). A cavity ring-down analyzer for measuring atmospheric levels of methane, carbon dioxide, and water. Appl. Phys. B.

[10] Ye J., Ma L.S., Hall J.L. (1998). Ultrasensitive detections in atomic and molecular physics: demonstration in molecular overtone spectroscopy. J. Opt. Soc. Am. B.

[11] Herriott D.R., Schulte H.J. (1965). Folded optical delay lines. Appl. Opt.

[12] Pavone F.S., Inguscio M. (1993). Frequency- and wavelength-modulation spectroscopies: Comparison of experimental methods using an AlGaAs diode laser. Appl. Phys. B.

[13] Cooper D.E., Warren R.E. (1987). Frequency modulation spectroscopy with lead-salt diode lasers: a comparison of single-tone and two-tone techniques. Appl. Opt.

[14] Silver J.A. (1992). Frequency-modulation spectroscopy for trace species detection: theory and comparison among experimental methods. Appl. Opt.

[15] Supplee J.M., Whittaker E.A., Lenth W. (1994). Theoretical description of frequency modulation and wavelength modulation spectroscopy. Appl. Opt.

[16] Wilson G.V.H. (1963). Modulation broadening of NMR and ESR line shapes. J. Appl. Phys.

[17] LI-COR Bioscience HP http://www.licor.com/env/Products/GasAnalyzers/li7700/7700.jsp.

[18] Humlicek J. (1982). Optimized computation of the Voigt and complex probability functions. J. Quant. Spectrosc. Radiat. Transfer.

[19] Schreier F. (1992). The Voigt and complex error function: A comparison of computational methods. J. Quant. Spectrosc. Radiat. Transfer.

[20] Rothman L.S., Jacquemart D., Barbe A., Benner D.C., Birk M., Brown L.R., Carleer M.R., Chackerian C., Chance K., Coudert L.H., Dana V., Devi V.M., Flaud J.-M., Gamache R.R., Goldman A., Hartmann J.-M., Jucks K.W., Maki A.G., Mandin J.-Y., Massie S.T., Orphal J., Perrin A., Rinsland C.P., Smith M.A.H., Tennyson J., Tolchenov R.N., Toth R.A., Auwera J.V., Varanasi P., Wagner G. (2005). The HITRAN 2004 molecular spectroscopic database. J. Quant. Spectrosc. Radiat. Transfer.

[21] Reid J., Labrie D. (1981). Second-harmonic detection with tunable diode lasers-Comparison of experiment and theory. Appl. Phys. B.

[22] Parkes A.M., Linsley A.R., Orr-Ewing A.J. (2003). Absorption cross-sections and pressure broadening of rotational lines in the 3ν_3_ band of N_2_O determined by diode laser cavity ring-down spectroscopy. Chem. Phys. Lett.

